# Recurrence after thymoma resection according to the extent of the resection

**DOI:** 10.1186/1749-8090-9-51

**Published:** 2014-03-19

**Authors:** Mi Kyung Bae, Seok Ki Lee, Ha Yan Kim, Seong Yong Park, In Kyu Park, Dae Joon Kim, Kyung Young Chung

**Affiliations:** 1 Department of Thoracic and Cardiovascular Surgery, Seoul National University, Bundang Hospital, Seoul National University College of Medicine, Seoul, Korea; 2 Department of Thoracic and Cardiovascular Surgery, Yonsei University College of Medicine, Seoul, Korea; 3 Biostatistics Collaboration Unit, Yonsei University College of Medicine, Seoul, Korea; 4 Department of Thoracic and Cardiovascular Surgery, Seoul National University Hospital, Seoul National University College of Medicine, Seoul, Korea

**Keywords:** Thymoma, Thymectomy, Recurrence

## Abstract

**Background:**

Complete resection of the thymus is considered appropriate for a thymoma resection because any remaining thymic tissue can lead to local recurrence. However, there are few studies concerning the extent of thymus resection. Therefore, we conducted a retrospective study to investigate whether recurrence following thymoma resection correlated to the extent of resection.

**Methods:**

Between 1986 and 2011, a total of 491 patients underwent resection of thymic epithelial tumors with curative intent. Of those, we excluded patients with an undetermined World Health Organization (WHO) histologic type, patients with type C thymoma, and patients who underwent incomplete resection (n = 21). The remaining 342 patients were reviewed retrospectively and compared recurrence according to the extent of resection.

**Results:**

Extended thymectomy was performed in 239 patients (69.9%) and limited thymectomy was performed 103 patients (30.1%). In the extended thymectomy group, 29 recurrences occurred, and in the limited thymectomy group, 10 recurrences occurred.

Comparing rates of freedom from recurrence between two groups, there was no significant statistical difference in total recurrence (*p* =0.472) or local recurrence (*p* =0.798). After matching patients by stage and tumor size, there was no significant difference in freedom from recurrence between the two groups (*p* = 0.162). Additionally, after adjusting for histologic type and MG, there was also no significant difference (*p* = 0.125) between groups.

**Conclusions:**

No difference in the rate of recurrence was observed in patients following limited thymectomy compared with extended thymectomy.

## Background

Complete resection of the thymus gland has been widely supported as part of thymoma resection because intrathymic metastasis or multifocal recurrence of thymoma can lead to local recurrence [1,2]. For the same reason, the International Thymic Malignancy Interest Group (ITMIG) suggests a complete thymectomy for patients without myasthenia gravis (MG) and extended thymectomy for patients with MG [3]. Thus, surgical resection of anything less than a complete thymectomy is considered inappropriate at this time except in the context of a clinical trial [3].

However, as detection of smaller thymomas has improved, and minimally invasive techniques are being more widely applied in thymoma resections, limited thymectomy is being considered for resections of non-invasive thymomas.

Furthermore, while many studies have shown that extended thymectomy is more effective for thymoma with MG [4,5], there is little evidence that mandates surgeons perform complete thymectomy for patients without MG.

At our institution, any patient with MG that is managed surgically undergoes extended thymectomy. However, surgical treatment for patients without MG does sometimes involve limited thymectomy, depending on the stage and size of the thymoma as well as the clinical judgment of the surgeon. This policy has been supported by the results of a report previously published by our institution [6], which found that there was no difference in survival between extended thymectomy and limited thymectomy (thymomectomy).

Following the proposal by ITMIG regarding the appropriate extent of resection, we questioned whether the extent of thymoma resection correlates with a significant difference in recurrence rates. This retrospective study was conducted in order to investigate whether the rate of recurrence is significantly different following thymoma resection relative to the extent of Resection.

## Methods

### Patients and definitions

Between January 1986 and December 2011, a total of 491 consecutive patients underwent surgery for resection of epithelial tumors of the thymus at our institute. Exclusions were made for patients with an undetermined World Health Organization (WHO) histologic type (n = 20), due to the unavailability of specimen slides or a completely infarcted tumor, patients with type C thymoma (n = 76), patients who only underwent an open biopsy or R2 resection (n = 21; R2 resection indicated macroscopic residual tumor tissue) and patients who underwent R1 resection (n = 32; R1 resection indicated microscopic residual tumor tissue). The remaining 342 patients who underwent R0 resection (no residual tumor tissue) with curative intent were reviewed retrospectively. The reason we included only R0 resection was that ITMIG recommended freedom-from-recurrence as the best measurement of clinical outcome for patients only who have undergone an R0 resection. Of the patients, 239 patients (69.9%) underwent extended thymectomy and 103 (30.1%) underwent limited thymectomy. The thymomas were classified into histological types A, AB, B1, B2 and B3, according to the WHO classification system [7]. When a tumor exhibited mixed histologic types, the tumor was classified using the most histologically aggressive type observed. For example, when the tumor had both B2 and B3 components, the tumor was classified as type B3. Tumor stage was classified into I, IIa, IIb, III, IVa, IVb, following the Masaoka-Koga classification system [8]. Recurrence was divided into three categories (local, regional and distant recurrence) according to the definition proposed by ITMIG [9]. The Institutional Review Board of Yonsei University College of Medicine approved this retrospective study. The need for individual consent of patients whose records were evaluated was waived because individuals were not identified in the study.

### Surgery

Extended thymectomy was defined as removal of the contiguous right and left mediastinal pleura, mediastinal and pericardiophrenic fatty tissues and dissection of the aorta-pulmonary window in addition to complete resection of the thymus and thymoma [3]. Limited thymectomy was defined as resection of the thymoma with the surrounding thymus and fatty tissue, leaving residual thymic tissue. The definition was the same as those used by Onuki et al. [10]. When limited thymectomy was performed, a “no-touch” technique was used to avoid disruption of the capsule. While resecting the thymoma, the tumor was extracted along with surrounding thymus and fatty tissue, taking care not to rupture the mass and spill tumor contents.

### Adjuvant therapy

Our strategies for adjuvant therapy were as follows: no adjuvant therapy for stage I thymomas, radiotherapy for invasive or incompletely resected thymomas, and chemotherapy (with or without radiotherapy) for Masaoka stage IV thymomas. However, because this strategy was not standardized for the entire study period, the strategy for adjuvant therapy was patient-specific according to each surgeon’s preference.

Adjuvant chemotherapy was generally performed according to the ADOC regimen (doxorubicin, cisplatin, vincristine, and cyclophosphamide) for a total of 6 cycles every 3–4 weeks. Radiotherapy was applied at the median dose of 5,040 cGy (180 cGy/fraction, range, 4500 to 6300 cGy) for 5–6 weeks.

### Follow up

All patients were followed up at the outpatient clinic at 3- or 6-month intervals, depending on stage, for two years, and then yearly for the following 3 years or more, at the discretion of the surgeon. A physical examination and chest radiograph were performed upon each visit to the outpatient clinic. Computed tomography (CT) scan of the chest was obtained at 6-month intervals for the first 2 years and yearly for the following 3 years or more. Patients were also followed at neurology or oncology clinics as needed for further medical management. For nine patients who did not attend follow-up appointments, data was obtained by direct telephone contact. Follow-up for this study was complete up to August 2012.

### Statistical analysis

This study was designed with statisticians in order to compare the difference of recurrence between extended thymectomy group and limited thymectomy group among our patients.

Because stage and tumor size could influence the decision of extent of resection, we matched stage and tumor size (±0.5 cm). In addition, because the presence of MG or histologic type could influence the recurrence after resection, freedom from recurrence was calculated after adjusting for the influence of MG and histologic type using Cox-regression. Namely, Cox regression analysis was performed after the extent of resection, the presence of MG and histologic type were set as covariates.

An exact 1:1 matching analysis was performed to get a well-balanced comparison using the GREEDY algorithm and a macro developed by Kosanke and Bergstralh [11]. The statistician carried out the matching process using SAS version 9.2 (SAS Institute Inc., Cary, NC, USA). Before matching, Comparative analyses to identify differences in patient and tumor characteristics were performed using the χ^2^ test of Fisher’s exact test to analyze categorical variables, and independent *t-*test to analyze for continuous variables. After matching, comparative analyses were performed using McNemar test to analyze categorical variables, and paired *t-*test to analyze for continuous variables.

Freedom from recurrence was calculated using the Kaplan-Meier method, and statistical differences in recurrence were determined using the log-rank test. Recurrences of thymoma were considered as events. All deaths without recurrence were considered as censored observations. The recurrence-free period following resection was calculated from the date of resection to the date of last follow-up or diagnosis of recurrence. A *P*-value of less than 0.05 was considered statistically significant. All statistical analyses except matching analysis were performed using the Statistical Package for Social Science software version 18.0 for Windows (SPSS Inc. Chicago, Illinois, USA).

## Results

Patients in the extended thymectomy group were significantly younger, had a higher incidence of MG, had histologically more aggressive types (B2 or B3), had a higher proportion of operations requiring median sternotomy, had smaller tumors, and a more advanced Masaoka-Koga stage compared to those in the limited thymectomy group (Table 1).

**Table 1:** Clinical and surgical characteristics of patients

**Variables**	**Extended thymectomy**	**Limited thymectomy**	***P value***
	**(n = 239)**	**(n = 103)**	
Age, mean ± SD	45.4 ± 12.10	49.7 ± 13.69	0.005
Sex			0.196
Male	123 (51.5)	61 (59.2)	
Female	116 (48.5)	42 (40.8)	
MG			< 0.0001
No	53 (22.2)	92 (89.3)	
Yes	186 (77.8)	11 (10.7)^a^	
WHO histologic type			< 0.0001
A	8 (3.3)	15 (14.6)	
AB	36 (15.1)	42 (40.8)	
B1	44 (18.4)	14 (13.6)	
B2	59 (24.7)	19 (18.4)	
B3	92 (38.5)	13 (12.6)	
Operative approach			< 0.0001
Median sternotomy	167 (69.9)	11 (10.7)	
Thoracotomy	13 (5.4)	32 (31.1)	
VATS	55 (23.0)	55 (53.4)	
Clamshell	4 (1.7)	-	
Cervicotomy	-	2 (1.9)	
Robotic	-	3 (2.9)	
Tumor size, mean ± SD	5.7 ± 3.13	6.9 ± 2.95	0.001
Masaoka –Koga stage			0.005
I	110 (46.0)	66 (64.1)	
IIa	50 (20.9)	21 (20.4)	
IIb	33 (13.8)	12 (11.7)	
III	34 (14.2)	4 (3.9)	
IVa	10 (4.2)	-	
IVb	2 (0.8)	-	
Adjuvant therapy			< 0.0001
None	113 (47.3)	79 (76.7)	
Radiotherapy	96 (40.2)	22 (21.4)	
Chemotherary	6 (2.5)	0 (0.0)	
Chemoradiation	24 (10.0)	2 (1.9)	
Follow-up time, mean ± SD	79.6 ± 59.49	81.2 ± 65.23	0.830

^a^post-thymectomy myasthenia gravis.

**Figure 1:**
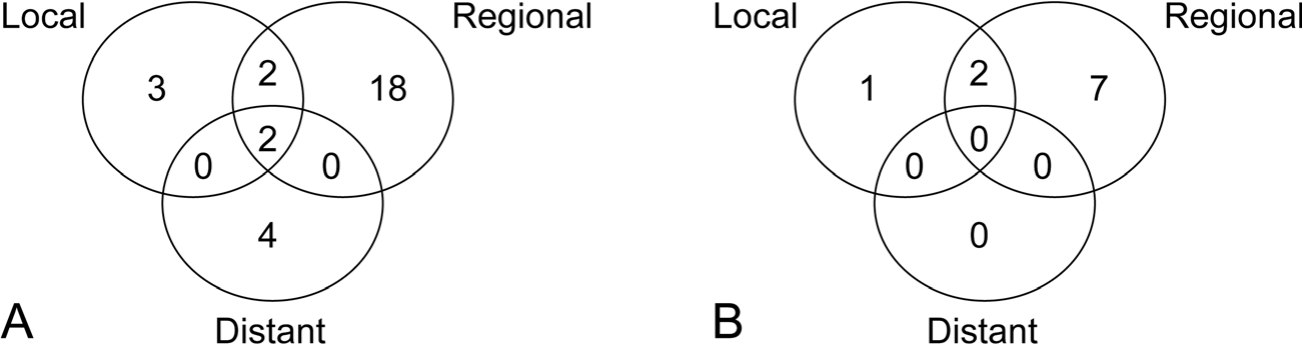
**The pattern of recurrence. A.** The pattern of recurrence of patients before matching. **B.** The pattern of recurrence of patients after matching by stage and tumor size.

The median follow up time was 63.0 months (range, 1 to 255 months). The median time to recurrence was 52 months (range, 6 to 234 months). Recurrence was observed in 39 (11.4%) of the 342 patients. The recurrence rate was 12.1% (29/239) in the extended thymectomy group and 9.7% (10/103) in the limited thymectomy group. Regional recurrence was the most common in both groups. The local recurrence rate including overlapped recurrence was 24% and 30% in the extended and limited thymectomy groups, respectively. Distant recurrence was discovered in six patients in the extended thymectomy group: two lung metastases, one liver, one abdominal lymph node, one lung + regional + local, and one bone + regional + local recurrence. The pattern of recurrence is shown in Figure 1.

**Figure 2:**
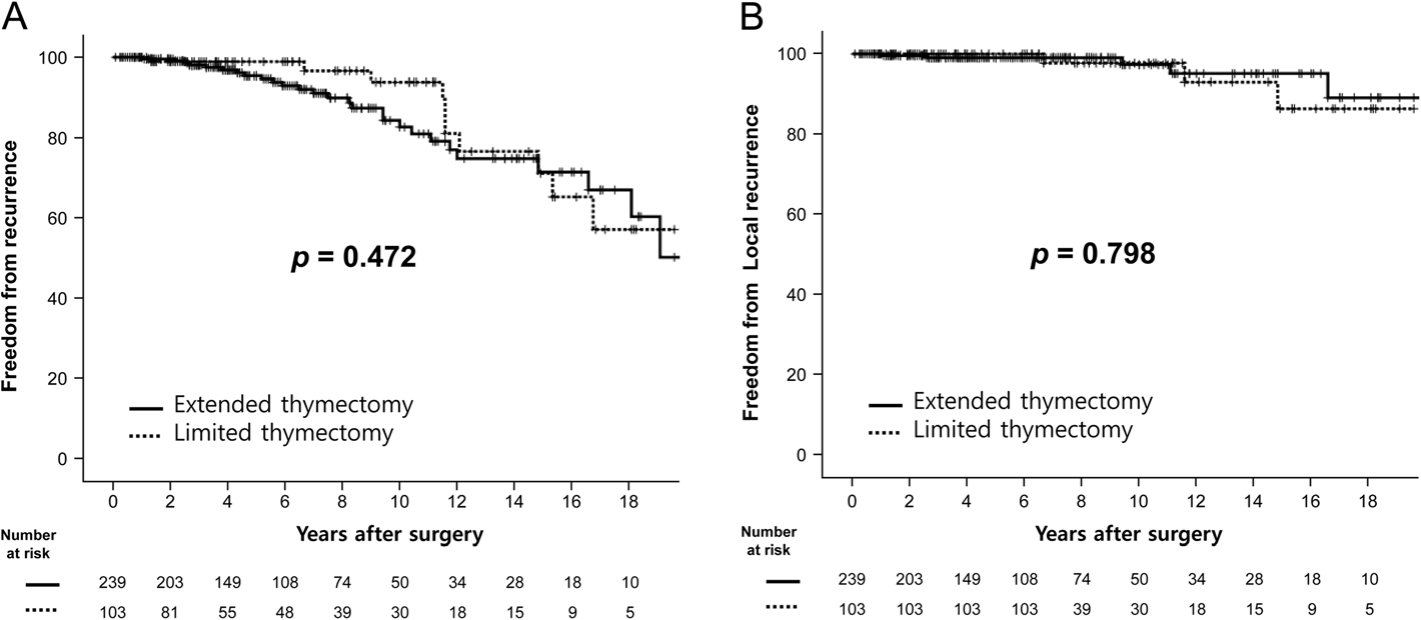
**Freedom from recurrence and freedom from local recurrence after thymoma resection. (A)** The 5-, 10- and 15-year freedom from recurrence rates were 95.4%, 82.7% and 71.4% in the extended thymectomy group, 98.9%, 93.8% and 71.1% in the limited thymectomy group, *p* = 0.472). **(B)** The 5-, 10- and 15-year freedom from local recurrence rates were 99.0%, 97.8% and 95.1% in the extended thymectomy group, 100.0%, 97.7% and 86.4% in the limited thymectomy group, *p* = 0.798).

Regarding freedom from recurrence, there was no significant statistical difference observed between the extended and limited thymectomy groups, in terms of total recurrence (*p* =0.472) as well as local recurrence (*p* =0.798) (Figure 2).

**Figure 3:**
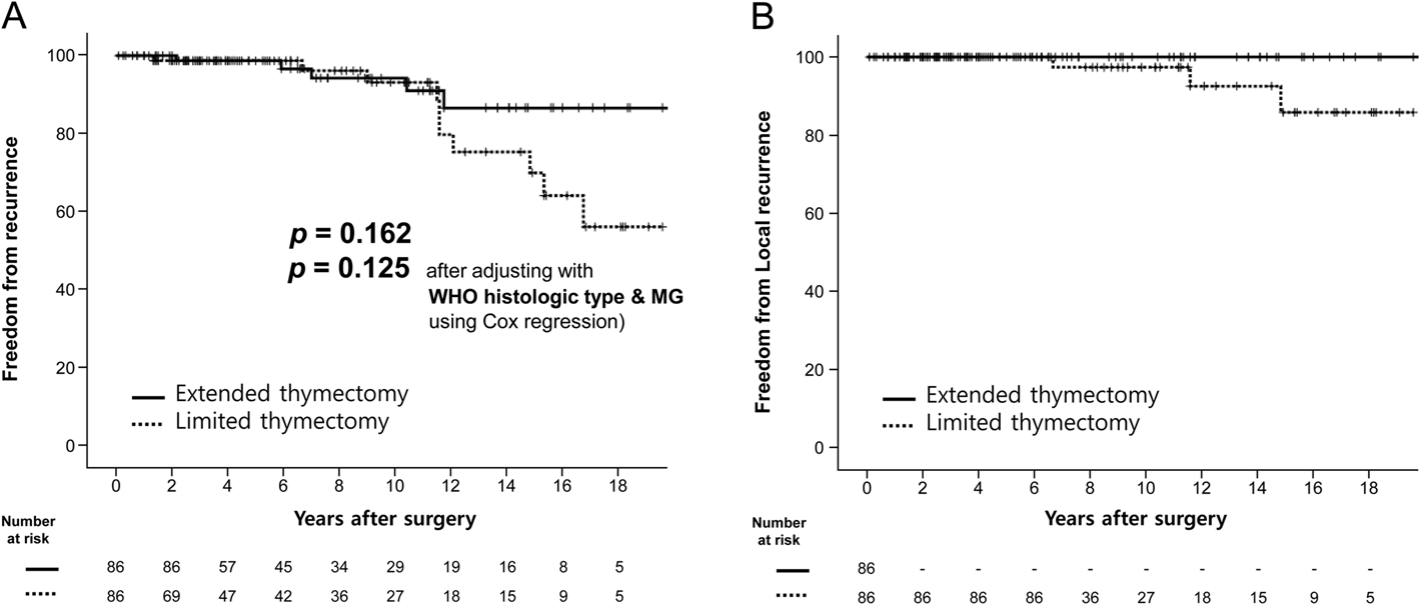
**Freedom from recurrence and freedom from local recurrence after 1:1 exact matching by size and stage of tumor. (A)** The 5-, 10- and 15-year freedom from recurrence rates were 98.7%, 94.2% and 86.6% in the extended thymectomy group, 98.7%, 93.1% and 70.0% in the limited thymectomy group, *p* = 0.162; *p* = 0.125 after adjusting with WHO histologic type and the presence of myasthenia gravis). **(B)** Freedom from local recurrence after 1:1 exact matching. There was no local recurrence in the extended thymectomy group. The 10- and 15-year freedom from recurrence rates were 97.4% and 85.9% in the limited thymectomy group.

Through exact 1:1 matching, 86 patients were selected from each group. Despite the matching protocol, the extended thymectomy group showed a higher incidence of MG and a more aggressive histologic type (Table 2). After matching, there was no significant difference in regards to freedom from recurrence between the two groups (*p* = 0.162). Furthermore, after adjusting for the influence of MG and histologic type using Cox-regression, there was also no significant difference (*p* = 0.125) (Figure 3A). Because there was not one case of local recurrence in the extended thymectomy group, it is not possible to report a statistical value for freedom from local recurrence (Figure 3B).

**Table 2:** Clinical and surgical characteristics of patients after 1:1 exact matching by size and stage of tumor

**Variables**	**Extended thymectomy**	**Limited thymectomy**	***P value***
	**(n = 86)**	**(n = 86)**	
Age, mean ± SD	45.3 ± 12.14	49.7 ± 13.60	0.027
Sex			0.284
Male	43 (50.0)	51 (59.3)	
Female	43 (50.0	35 (40.7)	
MG			< 0.0001
No	22 (25.6)	76 (88.4)	
Yes	64 (74.4)	10 (11.6)^a^	
WHO histologic type			0.001
A	6 (7.0)	12 (14.0)	
AB	15 (17.4)	34 (39.5)	
B1	20 (23.3)	12 (14.0)	
B2	17 (19.8)	17 (19.8)	
B3	28 (32.6)	11 (12.8)	
Operative approach			< 0.0001
Median sternotomy	61 (70.9)	8 (9.3)	
Thoracotomy	8 (9.3)	27 (31.4)	
VATS	16 (18.6)	47 (54.7)	
Clamshell	1 (1.2)	-	
Cervicotomy	-	2 (2.3)	
Robotic	-	2 (2.3)	
Tumor size, mean ± SD	6.4 ± 2.67	6.4 ± 2.67	0.943
Masaoka –Koga stage			>0.999
I	51 (59.3)	51 (59.3)	
IIa + IIb	31 (36.1)	31 (36.1)	
III	4 (4.6)	4 (4.6)	
IVa + IVb	-	-	
Adjuvant therapy			0.069
None	50 (58.1)	64 (74.4)	
Radiotherapy	30 (34.9)	21 (24.4)	
Chemotherary	2 (2.3)	0 (0.0)	
Chemoradiation	4 (4.7)	1 (1.2)	
Follow-up time, mean ± SD	94.5 ± 65.35	85.6 ± 67.92	0.386
**Pattern of recurrence**			
Local	0	1	
Local + regional	0	2	
Regional	3	7	
Distant	2	0	

^a^post-thymectomy myasthenia gravis.

## Discussion

With respect to recurrence after thymoma resection, there was no significant difference observed between recurrences of the groups in this study. However, our results do not necessarily mean that limited thymectomy is appropriate for thymoma resection. There are issues other than risk of recurrence that should be considered in order to determine the extent of resection. Nevertheless, this study might have a significance in that there have been few studies comparing recurrence according to the extent of thymus resection, apart from interpretation of our result or propriety of our policy for thymoma resection. Because thymomas are slow growing and relatively rare, most studies on this subject have been retrospective, single institution, observational studies that report results over an extended time period. Thus, although the National Comprehensive Cancer Network (NCCN) guidelines recommend the proper extent of thymoma resection as complete excision of the lesion with total thymectomy [12], these recommendations are based on lower-level evidence because there have been no properly controlled prospective studies in this area. Accordingly, many institutions treat patients according to their own protocols or using individual surgeon’s clinical experience and judgment. In particular, the extent of resection of thymus differs at the discretion of the surgeon. According to Kondo et al. [13], among patients with thymoma and MG, 74.8% (190/259) underwent extended thymectomy, 23.2% (59/259) underwent total thymectomy (thymothymectomy) and 2.0% (5/259) underwent limited thymectomy (resection of the tumor and a portion of the thymus). Among patients who had thymoma without MG, 37.7% (273/770) underwent extended thymectomy, 43.4% (315/770) underwent total thymectomy and 18.9% (137/770) underwent limited thymectomy. In our institution, all patients with MG underwent extended thymectomy except 11 who were diagnosed with MG following surgery (post-thymectomy MG). Among patients without MG, 36.6% (53/145) underwent extended thymectomy and 63.4% (92/145) underwent limited thymectomy.

It is accepted that surgical resection is the treatment of choice for thymoma and complete resection is the primary objective [14-18]. The completeness of resection implies removal of the tumor along with any adjacent tissues that appear to be invaded by a thymoma [19], but does not necessitate complete thymus resection. Thus, our institution strives to perform complete thymoma resections by that definition. Even in limited thymectomy cases, we avoid tumor spillage by resecting the thymoma along with the surrounding fatty and thymic tissues, without exposing the tumor at any point, using the so-called “no-touch” technique.

Similar to other reports, our data has confirmed that completeness of resection by the above definition is an important prognostic factor for recurrence after thymoma resection [20]. However, contrary to our expectation, the extent of resection is not significantly correlated with risk for recurrence. Similar results have been reported in a few other studies, that there is no difference in prognosis based on the extent of resection. Nakagawa et al. reported that there was no difference in survival between patients undergoing complete thymectomy (n = 71) and those undergoing thymomectomy (n = 55) [18]. Onuki et al. reported that, compared with complete thymectomy, limited thymectomy is not inferior for treatment of stage I or II thymoma in regards to disease-free and overall survival, post-operative onset of MG, and the incidence of multiple lesions [10]. Sakamaki et al. reported that limited thymectomy by a unilateral thoracoscopic approach was associated with better short-term outcomes than extended thymectomy by infrasternal mediastinal approach [11]. In that series, they also reported that no recurrence has been detected within the mean follow-up period of 48 months. On the contrary, Murakawa et al., reported the results from a 43-year period of surgical treatment for thymoma [21], suggesting that extended thymectomy is the preferred treatment for stage I thymoma. They observed that 4 of 64 patients with stage I thymoma developed recurrence, and all patients with recurrence had been managed with thymomectomy only. Additionally, another report by that group described three cases of local recurrence out of 162 patients that underwent extended thymectomy. These three were of advanced stage (III or IVa) thymoma initially, whereas there were no local recurrences observed in patients with early stage (I or II) disease [22]. Wang et al. reported that patients who underwent complete thymectomy (n = 16) had a better survival rate than those who underwent total tumor resection (n = 18) [23].

Because many of the results reported are conflicting and come from single-institution retrospective studies with various lengths of follow-up, it is difficult to conclude how recurrence risk is affected by the amount of thymic tissue permitted to remain during surgery.

Our study, like many previously, is a retrospective study performed at a single institution over a long period of time. Thus, our results should be interpreted cautiously, and it cannot be supported as reflecting strong evidence on this subject. Even though differences between extended thymectomy and limited thymectomy were not statistically significant, the graph after matching demonstrated an obvious difference in freedom from recurrence after more than ten years of surgery. Moreover, no case of local recurrence was observed in the extended thymectomy group after matching. Because recurrence takes a long time to develop with early-stage thymoma, this difference is worthy of attention and requires investigation over a much longer period of time. We expect that prospective studies will be conducted similar to the one currently ongoing by the Japanese Association for Research on the Thymus, a clinical trial titled “Phase II Study of Thymomectomy for Thymoma Localized in the Thymus” [24]. Hopefully, studies such as this will help provide clearer answers to the aforementioned questions.

In order to determine an appropriate extent of resection, there are several issues to be considered in addition to risk of recurrence.

First, when limited thymectomy is performed, one issue is how to guarantee a sufficient margin while achieving complete resection. Transcapsular perithymic invasion can be subtle and is often not grossly appreciable by the surgeon peri-operatively [25]. Thus, even when resection is performed to include the surrounding thymus and fatty tissue, it is unclear how much tissue in addition to thymoma should be resected to ensure complete resection. In many of these limited thymectomy cases, despite a small and seemingly well-encapsulated thymoma, there is a concern of incomplete resection should there be microscopic invasion into the surrounding tissue. Our data suggest, however, that limited resection and incomplete resection are not associated. There were eleven patients who were classified as an R1 resection because the tumor was abutting or involving the resection margin, even considering their diagnosis of early stage thymoma (IIa; n = 10, IIb; n = 1); these patients were excluded from the present study, and only R0 resections were included. Among those patients, eight underwent extended thymectomy by a median sternotomy approach, two patients had limited thymectomy by thoracotomy and one patient had a limited thymectomy by cervicotomy. From these cases, we can infer cautiously that size and location of the thymoma, as well as the operative approach used, may be associated with completeness of resection, rather than extent of thymus resection being an indicator.

Secondly, postoperative MG is also important issue related to the extent of resection. The rate of postoperative MG in patients without MG at the time of thymoma resection is between 1.0 to 4.6% [26]. In our series, eleven patients (3.2%) developed MG postoperatively, and each one had undergone limited thymectomy. From this perspective, any amount of remaining thymic tissue following surgery, or presence of ectopic thymus, can lead to MG following thymoma resection. However, further studies will be needed to confirm the hypothesis that completes resection of thymus can prevent postoperative MG.

Thirdly, multifocal development of thymoma in remnant thymic tissue is also a concern related to the extent of thymectomy. Previous studies have reported that multiple lesions of thymoma were known in 1.0 to 3.1% of all patients undergoing resection [1,2]. There were no cases of multifocal thymoma in our patients.

In order to get find answers to these concerns, and hopefully provide a solution to the questions of the appropriate extent of resection of thymoma, well-controlled prospective studies will be necessary. We expect that, in the future, international collaborative data may be supported by ITMIG, and randomized-controlled prospective studies will provide more defined protocols for the surgical management of thymomas.

There are limitations in our study. First, we used matching analysis using stage and tumor size because these factors affect the surgical procedure. However, the presence of MG is also a key factor that might affect the surgical procedure. We therefore presume that it would be rational to perform comparative studies solely in patients without MG, as proposed by Tseng et al. [27]. At our medical insitution, however, few patients with early-stage thymoma without MG have undergone extended thymectomy. This explains the reason why we failed to evaluate the patients without MG. Thus, we could not match the presence of MG. Instead, there was a notable difference in the presence of MG between the two groups. We therefore adjusted the presence of MG, a key factor that might affect the recurrence, for the Cox analysis. Our statistical methods remain problematic, however, our results might be of significance in that there are few comparative studies about the recurrence depending on the extent of thymic resection. Second, this study was a retrospective study performed at a single institution covering a long period, thus, the results might be influenced by intrinsic bias. However, this problem would be in almost all studies about thymoma because thymoma is slow-growing, rare tumor. Third, our surveillance might underestimate the recurrence rate because not every patient takes a regular chest CT after more than five years. However, it would not be problematic to compare between the two groups because the limitation might be consistently applied to both groups. This problem is also attributed that there has been no standard surveillance in thymoma so far. We expect that international collaborative data and randomized-controlled prospective studies could provide a standard surveillance.

## Conclusion

In summary, limited thymectomy for thymoma resection does not show a higher rate of recurrence than extended thymectomy. However, properly controlled prospective studies are needed to confirm the validity and reliability of limited thymectomy for thymoma resection.

## Competing interests

The authors declare that they have no competing interests.

## Authors’ contributions

MKB participated in all sections of the manuscript. SKL and SYP contributed to collection and analysis of data. HYK carried out the statistical analysis. IKP and DJK participated in study design and interpretation of data. KYC supervised all the work for the manuscript and helped to draft the manuscript. All authors read and approved the final manuscript.

## References

[B1] Mori T Nomori H Ikeda K Yoshioka M Kobayashi H Iwatani K Yoshimoto K Iyama K Three cases of multiple thymoma with a review of the literature Jpn J Clin Oncol 2007 9 146 149 10.1093/jjco/hyl147 17337514

[B2] Suzuki H Yoshida S Hiroshima K Nakatani Y Yoshino I Synchronous multiple thymoma: report of three cases Surg Today 2010 9 456 459 10.1007/s00595-009-4080-z 20425550

[B3] Toker A Sonett J Zielinski M Rea F Tomulescu V Detterbeck FC Standard terms, definitions, and policies for minimally invasive resection of thymoma J Thorac Oncol 2011 9 S1739 S1742 21847056 10.1097/JTO.0b013e31821ea553

[B4] Masaoka A Monden Y Comparison of the results of transsternal simple, transcervical simple, and extended thymectomy Ann N Y Acad Sci 1981 9 755 765 10.1111/j.1749-6632.1981.tb33773.x 6951498

[B5] Jaretzki A 3rd Bethea M Wolff M Olarte MR Lovelace RE Penn AS Rowland L A rational approach to total thymectomy in the treatment of myasthenia gravis Ann Thorac Surg 1977 9 120 130 10.1016/S0003-4975(10)63720-4 879895

[B6] Kim DJ Yang WI Choi SS Kim KD Chung KY Prognostic and clinical relevance of the world health organization schema for the classification of thymic epithelial tumors: a clinicopathologic study of 108 patients and literature review Chest 2005 9 755 761 10.1378/chest.127.3.755 15764754

[B7] Rosai J Sobin L Histological Typing of Tumors of the Thymus: International Histological Classification of Tumors 1999 2 New York, Berlin: Springer

[B8] Koga K Matsuno Y Noguchi M Mukai K Asamura H Goya T Shimosato Y A review of 79 thymomas: modification of staging system and reappraisal of conventional division into invasive and non-invasive thymoma Pathol Int 1994 9 359 367 8044305 10.1111/j.1440-1827.1994.tb02936.x

[B9] Huang J Detterbeck FC Wang Z Loehrer PJ Sr Standard outcome measures for thymic malignancies J Thorac Oncol 2010 9 2017 2023 10.1097/JTO.0b013e3181f13682 20978450

[B10] Onuki T Ishikawa S Iguchi K Goto Y Sakai M Inagaki M Yamamoto T Onizuka M Sato Y Ohara K Sakakibara Y Limited thymectomy for stage I or II thymomas Lung Cancer 2010 9 460 465 10.1016/j.lungcan.2009.08.001 19717204

[B11] Kosanke J Bergstralh E Match One or More Controls to Cases using the GREEDY Algorithm 2004 Mayo Clinic College of Medicine: Rochester, MN

[B12] National Comprehensive Cancer network (NCCN) Guidelines Available at http://www.nccn.org/professionals/physician_gls/f_guidelines.asp

[B13] Kondo K Monden Y Thymoma and myasthenia gravis: a clinical study of 1,089 patients from Japan Ann Thoracic Surg 2005 9 219 224 10.1016/j.athoracsur.2004.06.090 15620947

[B14] Kondo K Monden Y Therapy for thymic epithelial tumors: a clinical study of 1,320 patients from Japan Ann Thorac Surg 2003 9 878 884 discussion 884–875 10.1016/S0003-4975(03)00555-1 12963221

[B15] Limmer KK Kernstine KH Minimally invasive and robotic-assisted thymus resection Thorac Surg Clin 2011 9 69 83 vii 10.1016/j.thorsurg.2010.08.005 21070988

[B16] Falkson CB Bezjak A Darling G Gregg R Malthaner R Maziak DE Yu E Smith CA McNair S Ung YC Evans WK The management of thymoma: a systematic review and practice guideline J Thorac Oncol 2009 9 911 919 10.1097/JTO.0b013e3181a4b8e0 19557895

[B17] Detterbeck FC Parsons AM Thymic tumors Ann Thorac Surg 2004 9 1860 1869 10.1016/j.athoracsur.2003.10.001 15111216

[B18] Nakagawa K Asamura H Matsuno Y Suzuki K Kondo H Maeshima A Miyaoka E Tsuchiya R Thymoma: a clinicopathologic study based on the new world health organization classification J Thorac Cardiovasc Surg 2003 9 1134 1140 10.1016/S0022-5223(03)00798-0 14566259

[B19] Haniuda M Miyazawa M Yoshida K Oguchi M Sakai F Izuno I Sone S Is postoperative radiotherapy for thymoma effective? Ann Surg 1996 9 219 224 10.1097/00000658-199608000-00016 8757387 PMC1235345

[B20] Bae MK Lee CY Lee JG Park IK Kim DJ Yang WI Chung KY Predictors of recurrence after thymoma resection Yonsei Med J 2013 9 875 882 10.3349/ymj.2013.54.4.875 23709420 PMC3663243

[B21] Murakawa T Nakajima J Kohno T Tanaka M Matsumoto J Takeuchi E Takamoto S Results from surgical treatment for thymoma. 43 years of experience Jpn J Thorac Cardiovasc Surg 2000 9 89 95 10.1007/BF03218097 10769987

[B22] Sakamoto M Murakawa T Konoeda C Inoue Y Kitano K Sano A Fukayama M Nakajima J Survival after extended thymectomy for thymoma Eur J Cardiothorac Surg 2012 9 623 627 10.1093/ejcts/ezr026 22011779

[B23] Wang LS Huang MH Lin TS Huang BS Chien KY Malignant thymoma Cancer 1992 9 443 450 10.1002/1097-0142(19920715)70:2<443::AID-CNCR2820700212>3.0.CO;2-T 1617594

[B24] Phase II Study of Thymomectomy for Thymoma Localized in the Thymus Available at http://upload.umin.ac.jp//cgi-open-bin/ctr/ctr.cgi?function=history&action=list&type=summary&recptno=R000000743&language=E

[B25] Wright CD Management of thymomas Crit Rev Oncol Hematol 2008 9 109 120 10.1016/j.critrevonc.2007.04.005 17570676

[B26] Kondo K Monden Y Myasthenia gravis appearing after thymectomy for thymoma Eur J Cardiothorac Surg 2005 9 22 25 10.1016/j.ejcts.2005.03.039 15935686

[B27] Tseng YC Hsieh CC Huang HY Huang CS Hsu WH Huang BS Huang MH Hsu HS Is thymectomy necessary in nonmyasthenic patients with early thymoma? J Thorac Oncol 2013 9 952 958 10.1097/JTO.0b013e31828cb3c2 23594467

